# Outcomes of Vertical Expandable Prosthetic Titanium Ribs in Children With Early-Onset Scoliosis Secondary to Cerebral Palsy

**DOI:** 10.7759/cureus.13690

**Published:** 2021-03-04

**Authors:** Randa Elmallah, Travis Fortin, Josny Thimothee, Hamdi Sukkarieh, Patrick B Wright, M. Wade Shrader, Jaysson Brooks

**Affiliations:** 1 Orthopaedic Surgery, University of Mississippi Medical Center, Jackson, USA; 2 Orthopaedics, Boonshoft School of Medicine, Wright State University, Dayton, USA; 3 Pediatric Orthopaedic Surgery, Children's Hospital of Mississippi, Jackson, USA; 4 Orthopaedic Surgery, Nemours Children's Hospital/Alfred I. duPont Hospital for Children, Wilmington, USA; 5 Pediatric Orthopaedic Surgery, Children’s Hospital of Mississippi, Jackson, USA

**Keywords:** veptr, cerebral palsy, scoliosis, neuromuscular scoliosis

## Abstract

Purpose

Patients who have neuromuscular scoliosis, such as cerebral palsy (CP), often develop spinal deformities that negatively impact quality of life. The vertical expandable prosthetic titanium rib (VEPTR) was designed for thoracic insufficiency syndrome (TIS), but it has also been utilized in patients with CP with restrictive lung disease and spine deformity. Few studies report on VEPTRs in neuromuscular scoliosis; however, none reports on their utilization specifically in patients with CP. Our purpose was to assess if VEPTRs can improve spinal deformity and TIS in these patients.

Methods

A retrospective chart review was performed of all patients with CP and scoliosis treated with a VEPTR between 2008 and 2017. Eight patients were eligible for this study. The mean follow-up was four years. Outcomes evaluated were Cobb angle, pelvic obliquity, space available for lung ratio (SAL), T1-S1 height, and complication rates. A p-value of less than 0.05 was used for statistical significance.

Results

There were significant postoperative improvements in Cobb angle, pelvic obliquity, and T1-S1 height, but no statistical difference in SAL. Prior to final fusion, the mean number of VEPTR lengthening procedures was 3. The mean time from index surgery to final fusion was 3.7 years. The most common complications were infection (62.5%) and wound dehiscence (25%). Only 25% of patients did not have a complication.

Conclusion

VEPTRs demonstrated significant improvement in almost all parameters and may be valuable in improving TIS in patients with CP. The complication and reoperation rates were similar to those of VEPTRs used for other pathological conditions.

## Introduction

Early-onset scoliosis in patients who have cerebral palsy (CP) presents treatment challenges to pediatric orthopedic surgeons. Compared to idiopathic scoliosis, some curves in children with CP tend to start at an earlier age and progress more rapidly, resulting in more severe deformities. In these skeletally immature patients, non-operative options are often ineffective at slowing the progression of spinal deformity [1], and definitive spinal fusion at an early age may halt spine and thoracic cavity growth.

In addition, a significant concern in this patient population is congenital chest wall deformities resulting in thoracic insufficiency syndrome (TIS), which consequently stunts normal pulmonary development. In the early 2000s, Campbell et al. introduced the use of rib-based distraction rods to treat complex spinal deformities that resulted in TIS [2,3]. This system was named the vertical expandable prosthetic titanium rib (VEPTR) and was designed for skeletally immature patients with various degrees of impaired lung function who have or will likely soon develop TIS.

Previous studies on the use of VEPTRs in patients with neuromuscular and congenital scoliosis have shown promising improvements in curve correction and pulmonary function [4,5]; however, to date, there have been no studies in the literature examining the use of VEPTR constructs specifically in patients with CP. Therefore, the purpose of our study was to investigate the use of VEPTR constructs to treat spinal deformity and TIS in patients with CP.

## Materials and methods

Following approval by the Institutional Review Board, a retrospective chart review was conducted of all patients with neuromuscular scoliosis (NMS) who received VEPTR constructs between 2008 and 2017, yielding a total of 18 patients. Patients were then excluded if their NMS etiology was not CP and if they had less than two years of follow-up after getting a VEPTR construct. Seven patients were then excluded from the study due to a diagnosis other than CP. An additional three patients who met inclusion criteria of having both CP and VEPTR constructs were excluded due to a lack of adequate radiographs to allow for measurements. This left a total of eight patients (six females and two males) who were included in the study. All eight patients were of gross motor functional classification system (GMFCS) level V.

Radiographic and surgical outcomes evaluated were major Cobb angle, pelvic obliquity, T1-S1 height, lung height ratio, number of vertebrae spanned, number of ribs spanned, number of lengthening procedures needed, number of patients transitioned to final fusion, and the frequency of complications. Pelvic obliquity was measured as the acute angle between the following two lines: (1) a line drawn from the spinous process of the T1 vertebral body to the middle of the superior sacral plate and (2) a line drawn perpendicular to a line crossing the iliac crests [6]. This method has been shown to have the highest inter-observer and intra-observer reliability [7]. Radiographic measurements for the T1-S1 height were taken as the height from the center of the T1 superior endplate to the center of the S1 superior endplate. Lung height was measured as the distance from the apex of the first rib to the apex of the diaphragm ipsilaterally. The bilateral lung height measurement was then converted to a ratio of concave to convex lung height, which formed a value called the space available for lung ratio (SAL), which was used as a proxy for improvement in pulmonary function [6].

Statistical analysis

A paired two-sample t-test with a two-tailed distribution was used to determine statistical significance in the change between (1) preoperative and initial postoperative measurements and (2) preoperative and latest follow-up prior to removal for data collected on major Cobb angle, pelvic obliquity, T1-S1 height, and SAL. The analysis was performed using a p-value of <0.05 to determine statistical significance.

## Results

A total of eight patients were included in this study, with all initial VEPTR surgeries performed at a single children’s hospital. The major curve apex was to the left in six patients and to the right in two patients. The mean age for initial placement of the VEPTR construct was 5.9 years (range: 3.1 to 8.7 years). The mean length of follow-up between the index surgery and latest available follow-up was four years (range: 2 to 6.5 years).

Seven of eight patients received bilateral rib-to-pelvis instrumentation with the initial placement. One patient received only a right-sided VEPTR construct initially before eventually receiving a left-sided construct during a subsequent surgical procedure. Another patient received additional support from placement of a third VEPTR construct, which consisted of a left-sided rib-to-rib construct. The cohort had a mean of four lengthening procedures (range: one to six) prior to final removal (Table 1).

**Table 1:** Patient Data

Patient	Instrument Location	No. of Vertebrae Spanned	No. of Ribs Spanned	No. of Lengthening Procedures	Instrumented Levels on Definitive Fusion	Postoperative Complication
1	Rib-to-pelvis	16	11	1	T2-to-pelvis	Infection, hardware exposure, wound dehiscence
2	Rib-to-pelvis	14	9	1	T3-to-L4	Infection
3	Rib-to-pelvis	12	8	3	T4-to-pelvis	None
4	Rib-to-pelvis	16	11	5	T2-to-pelvis	Rod migration
5	Rib-to-pelvis	14	11	5	N/A	Infection, respiratory distress
6	Rib-to-pelvis	17	10	1	N/A	Infection
7	Rib-to-pelvis and rib-to-rib	19	11	6	N/A	Infection, hardware exposure, wound dehiscence
8	Rib-to-pelvis	15	10	6	N/A	None

Radiographic parameters

There were statistically significant differences in the mean Cobb angle between preoperative and initial postoperative visits (p < 0.05; Table 2).

**Table 2:** Radiographic Measurements

Assessment Parameters	Preoperative	Initial Postoperative	Latest Follow-Up	p-Value Preoperative vs. Initial Postoperative	p-Value Preoperative vs. Latest Follow-Up
Mean Cobb angle (degrees)	57.1 ± 11.6	39.3 ± 7.3	42.4 ± 14.5	0.00335	0.01266
Mean pelvic obliquity (degrees)	12.0 ± 6.9	6.9 ± 6.6	9.3 ± 6.6	0.01397	0.11116
Mean T1-S1 height (mm)	244.6 ± 31.9	323.2 ± 50.6	327.5 ± 53.3	0.0003	0.00084
Mean space-available-for-lung-ratio	1.0 ± 0.2	1.0 ± 0.2	1.1 ± 0.2	0.69256	0.47557

Although this magnitude of improvement was not sustained at the final follow-up, there remained a significant improvement in Cobb angle at the final follow-up when compared to preoperative values.

Similarly, mean pelvic obliquity significantly improved from preoperative to initial postoperative visit (p < 0.05; Table 2). However, this improvement was no longer significant at the final follow-up (p > 0.05). T1-S1 height improved significantly from preoperative to initial postoperative measurements and was sustained at the final follow-up (p < 0.05; Table 2). The SAL between concave and convex sides was not significantly improved at any follow-up point (p > 0.05; Table 2).

Final fusion

After the VEPTR construct was removed, four patients required definitive posterior spinal fusions (Table 1). The mean age for receiving a spinal fusion was 11.5 years (range: 10 to 13 years). The mean length of time between the index surgery and posterior spinal fusion was 3.4 ± 1.0 years (range: 2.4 to 4.4 years).

Complications

Postoperative complications associated with placement of the VEPTR constructs included recurrent infection, wound dehiscence, hardware exposure, rod migration, and respiratory distress (Table 1). The overall complication rate was 75% (six patients). Infection was the most common complication with a prevalence of 62.5% (five patients), each requiring surgical revision. Only two (25%) patients did not experience postoperative complications.

## Discussion

The use of VEPTRs to treat complex chest and spinal deformities has been instrumental in improving the quality of life in children with TIS; however, there has been no English language literature focusing on the use of VEPTRs in children only with the diagnosis of CP. We found that there were significant postoperative improvements in the patient’s Cobb angle, pelvic obliquity, and T1-S1 height; however, there was no significant improvement in SAL. In addition, we found a 75% complication rate, with the most common complications being infection (62.5%) and wound dehiscence (25%), likely due to a combination of repeat surgeries secondary to implant design as well as poor nutritional status and immune compromise in these patients.

Comparable literature to our study is sparse for children specifically with CP. However, several studies have assessed radiographic outcomes in patients who have received VEPTRs for both congenital and non-congenital anomalies. Ramirez et al. conducted a multi-center study looking at the use of rib-to-pelvis VEPTR constructs in 65 patients who had early-onset scoliosis from unspecified etiologies. The mean correction in Cobb angle between preoperative and final follow-up was 13 degrees, similar to the approximate mean of 15 degrees found in our study [8]. Pelvic obliquity, T1-S1 height, and SAL were not assessed. In contrast, White et al. evaluated the outcomes of spine-to-spine modified VEPTR constructs in 14 patients with either unspecified neuromuscular or congenital scoliosis. They found a mean Cobb angle improvement of 17 degrees, and that the T1-S1 height increased a mean of 36 mm and the SAL increased from 85% to 92%. In our study, we did not find any significant improvement in the SAL after treatment with a VEPTR construct [5]. In addition, their complication rate was also high at 43%, but not nearly as high as our complication rate at 75%. Bachabi et al. performed a direct comparison of VEPTRs and growing rods, although this was in 77 patients who had idiopathic early-onset scoliosis (50 patients had growing rod constructs, 22 had VEPTRs). At a mean follow-up of eight years, growing rod patients had a significantly greater correction in Cobb angle (50% versus 27%; p = 0.001) as well as greater gain in thoracic height compared to VEPTR patients (24% versus 12%; p = 0.024). VEPTR patients also had a greater progression in their kyphosis postoperatively [9].

When evaluating pulmonary function, it can be argued that radiographic measurements may not provide accurate surrogates for lung capacity, particularly as they are subjected to considerable inter-observer differences in measurement. Gadepalli et al. used pulmonary function tests and 3D reconstructions of thoracic CT scans to evaluate differences in lung volume and function following VEPTR surgery in 26 patients. There were no statistical differences in pulmonary function testings (PFTs) or volume on the CT scans at postoperative follow-up. However, their postoperative mean improvement in Cobb angles was only 6 to 8 degrees [10]. More recently, Tong et al. evaluated the use of free-breathing dynamic MRIs in 25 patients with TIS who were treated with VEPTRs [11]. Postoperatively, it was found that there were significant increases in right and left lung volume at end expiration (22.9% and 12%; p = 0.001) and increases in right and left lung tidal volume (55.3% and 43.8%; p = 0.001). Interestingly, when correlated with radiographic measurements, there was no significant association between right and left diaphragm tidal volumes and thoracic Cobb angles (p = 0.06 and 0.07, respectively). This demonstrates that the radiographic surrogates used to assess the thoracic cavity may not reflect true pulmonary function. Our study used SAL as a radiographic proxy for improvement in pulmonary function. Although there was a slight improvement in SAL, this was not statistically significant (p > 0.05). Furthermore, patients with CP typically have a difficult time in performing formal PFT either due to lack of physiological function or due to associated intellectual disability.

As stated above, the SAL may not be the most reliable means of assessing true function. In the absence of a significantly improved SAL, our patients may still experience an increase in pulmonary function following VEPTR placement, which cannot be adequately assessed radiographically. However, it may be hypothesized that an unchanged SAL may indicate that the VEPTR prevented further deterioration in pulmonary function that may have otherwise occurred, although this conclusion cannot be drawn with current data.

The overall rate of complication in our study was 75% (six patients), and only 25% (two patients) did not experience postoperative complications. Infection was the most common complication with a prevalence of 62.5% (five patients), each requiring surgical revision. However, the high complication profile in this patient population is well published and is often associated with the high morbidity of the procedure as well as the poor nutritional status of the host. Farley et al. conducted a case-control study evaluating the risk factors for infection in patients with idiopathic, congenital, or neuromuscular scoliosis treated with either VEPTR, growing rods, or posterior spinal fusion [12]. They found that of the 20 patients who developed deep surgical site infections, 14 had NMS and 6 had undergone VEPTR surgery. Increased Cobb angle, non-ambulatory status, and longer hospital stay were associated with significantly higher odds of infection. Crews et al. assessed the risk factors for surgical site infections in children who underwent VEPTR surgery. Each patient with an infection was matched to three control subjects. They found an overall infection rate of 8%. Surgical site infection was associated with male gender (OR: 3.5; 95% CI: 0.9-13.2), assisted feeding (OR: 4.5; 95% CI: 1-20.3), administration of preoperative antibiotics more than 30 minutes before surgery (OR: 6.9; 95% CI: 1.2-39), and intra-operative temperature less than 35 degrees Celsius (OR: 4.3; 95% CI: 0.8-23.7) [13]. Similarly, high complication rates have been found in children with CP who have had other types of growing rod constructs. McElroy et al. found that 19 (70%) of 27 patients experienced a complication following growing rod surgery, which predominantly included deep wound infections necessitating early fusion and instrument removal in a few patients, as well as rod exchange and rod fracture [6]. However, Bachabi et al. found a higher complication rate in VEPTRs when directly compared to growing rod surgery (82% versus 66%) and specifically in wound complications (41% versus 14%; p = 0.011) [9].

Our study has several limitations, the first being that unknown confounding variables may exist due to the retrospective nature of the data. The second limitation was the small number of included patients with complete information and radiographs, thus potentially introducing selection bias in our selection of patients. Despite these limitations, our study is the first to evaluate the use of VEPTR constructs in patients with CP.

## Conclusions

In conclusion, patients with scoliosis secondary to CP can successfully be treated with VEPTR constructs, which demonstrate improvements in radiographic outcomes, despite no corresponding significant improvements in TIS at the final follow-up. However, there are many other growing rod constructs available to these patients, including traditional growing rods and magnetically controlled growing rods. In addition, these alternative constructs offer improved complication profiles. Because of this, the authors no longer utilize VEPTR constructs in children with CP or other similar neuromuscular diagnoses.
